# Regulation of Wnt signaling by nociceptive input in animal models

**DOI:** 10.1186/1744-8069-8-47

**Published:** 2012-06-19

**Authors:** Yuqiang Shi, Subo Yuan, Bei Li, Jigong Wang, Susan M Carlton, Kyungsoon Chung, Jin Mo Chung, Shao-Jun Tang

**Affiliations:** 1 Department of Neuroscience and Cell Biology, University of Texas Medical Branch, Galveston, TX, 77555, USA

**Keywords:** Wnt, Synapse, Spinal dorsal horn, Pain, Nociception, β-catenin

## Abstract

**Background:**

Central sensitization-associated synaptic plasticity in the spinal cord dorsal horn (SCDH) critically contributes to the development of chronic pain, but understanding of the underlying molecular pathways is still incomplete. Emerging evidence suggests that Wnt signaling plays a crucial role in regulation of synaptic plasticity. Little is known about the potential function of the Wnt signaling cascades in chronic pain development.

**Results:**

Fluorescent immunostaining results indicate that β-catenin, an essential protein in the canonical Wnt signaling pathway, is expressed in the superficial layers of the mouse SCDH with enrichment at synapses in lamina II. In addition, Wnt3a, a prototypic Wnt ligand that activates the canonical pathway, is also enriched in the superficial layers. Immunoblotting analysis indicates that both Wnt3a a β-catenin are up-regulated in the SCDH of various mouse pain models created by hind-paw injection of capsaicin, intrathecal (i.t.) injection of HIV-gp120 protein or spinal nerve ligation (SNL). Furthermore, Wnt5a, a prototypic Wnt ligand for non-canonical pathways, and its receptor Ror2 are also up-regulated in the SCDH of these models.

**Conclusion:**

Our results suggest that Wnt signaling pathways are regulated by nociceptive input. The activation of Wnt signaling may regulate the expression of spinal central sensitization during the development of acute and chronic pain.

## Introduction

During the development of chronic pain, spinal neurons in the spinal cord dorsal horn (SCDH) become sensitized and hyper-active (termed central sensitization). A spectrum of neuronal and glial processes has been implicated in the establishment of central sensitization. For instance, in the spinal nerve ligation (SNL) and spared nerve injury (SNI) models of neuropathic pain, the central terminals of primary sensory neurons were reported to sprout [1-4]. This sprouting may increase inputs of nociceptive signals. Indeed, increased release of neurotransmitters or neuromodulators such as glutamate, substance P, prostaglandin E2 (PGE_2_) and calcitonin-gene related peptide (CGRP) were reported in animal pain models (reviewed in [5]). Another neuronal alteration associated with central sensitization is the expression of long-term potentiation (LTP) at the synapses in superficial layers of the SCDH, which is considered to be a critical synaptic mechanism underlying chronic pain [6,7] and a potential target for chronic pain therapy [8]. Furthermore, loss of inhibitory functions of GABAergic and glycinergic interneurons may contribute to enhanced pain sensitivity in chronic pain [9,10]. In addition to neuronal changes, more recent studies revealed an important role of glial cells, especially microglia and astrocytes, in central sensitization, and glia are emerging as a promising target for chronic pain treatment [11]. Activated microglia and astrocytes facilitate the development of central sensitization by releasing chemokines, cytokines and neurotrophins [12-14]. These factors can markedly enhance the excitability of neurons processing nociceptive input. For example, tumor necrosis factor-alpha (TNFα), a key proinflammatory cytokine, was shown to increase the frequency of excitatory postsynaptic currents (EPSCs) and N-methyl-D-aspartate (NMDA) currents in lamina II neurons by stimulating TNF receptor subtype-1 and 2 (TNFR1 and TNFR2) in an inflammatory pain model [15]. Despite significant progress in identifying various cellular processes that contribute to central sensitization and chronic pain, the molecular mechanisms by which the spectrum of cellular alterations is initiated and established remain poorly understood.

Secreted signaling proteins in the Wingless–Int (Wnt) family play essential roles in many aspects of neural development/plasticity [16,17], such as neurogenesis, axonal and dendritic branching, synapse formation, synaptic transmission and plasticity, and memory formation [18-33]. The synthesis and secretion of neuronal Wnt proteins are controlled by synaptic activity [28,33,34]. Three Wnt signaling pathways are well characterized, including the canonical Wnt/β-catenin pathway, the planar cell polarity (Wnt/PCP; a.k.a. Wnt/JNK) pathway and the Wnt/Ca^2+^ pathway [35]. In the canonical pathway, Wnts bind to the frizzled (Fz) receptors on plasma membranes. This interaction stimulates the disheveled (Dvl) scaffold protein, leading to the inhibition or disruption of the ‘destruction complex’, which contains glycogen synthase kinase-3β (GSK-3β), axin and adenomatous polyposis coli (APC). Consequently, the β-catenin protein is stabilized, accumulates in the cytoplasm, and is imported into the nucleus to activate the transcription of TCF/LEF (T-cell factor/lymphoid enhancer factor) target genes [36]. In hippocampal neurons, activation of NMDA receptors (NMDARs) causes β-catenin nuclear translocation from post-synaptic regions and activation of gene expression [37]. At synapses, β-catenin interacts with cadherin to regulate synaptic assembly, remodeling and plasticity [38,39]. In the PCP pathway, Wnt-bound Fz signals through Dvl and the GTPase RhoA to activate c-Jun amino (N)-terminal kinase (JNK) which regulates cytoskeleton dynamics and transcription [40-42]. JNK signaling plays important roles in central sensitization induced by inflammation and nerve injury [43-45]. One mechanism by which JNK signaling contributes to chronic pain is to regulate the expression of cytokines (e.g., IL-10, TNFα, IL-1β and IL-6) in the spinal glia cells [44]. In the Wnt/Ca^2+^ pathway, Fz activation leads to increased intracellular Ca^2+^, which thereby activates Ca^2+^-sensitive proteins such as protein kinase C (PKC) and calcium/calmodulin dependent protein kinase II (CaMK II) [46]. Both PKC and CaMKII play pivotal roles in central sensitization during the development of neuropathic and inflammatory pain [47-50]. Despite of the accumulation of suggestive evidence, the involvement of Wnt signaling in pathological pain has not been directly tested.

In this study, we report the spatial distribution of specific Wnt signaling proteins in mouse spinal cords and the regulated expression of the proteins in multiple pain models. Our results reveal the expression of Wnt signaling proteins in the superficial layers of the SCDH and the up-regulation of their expression in acute and chronic pain models. These findings indicate that Wnt signaling pathways may play a role in the regulation of central sensitization and chronic pain development.

## Results

### Spatial distribution of β-catenin and Wnt3a in the mouse SCDH

**Figure 1:**
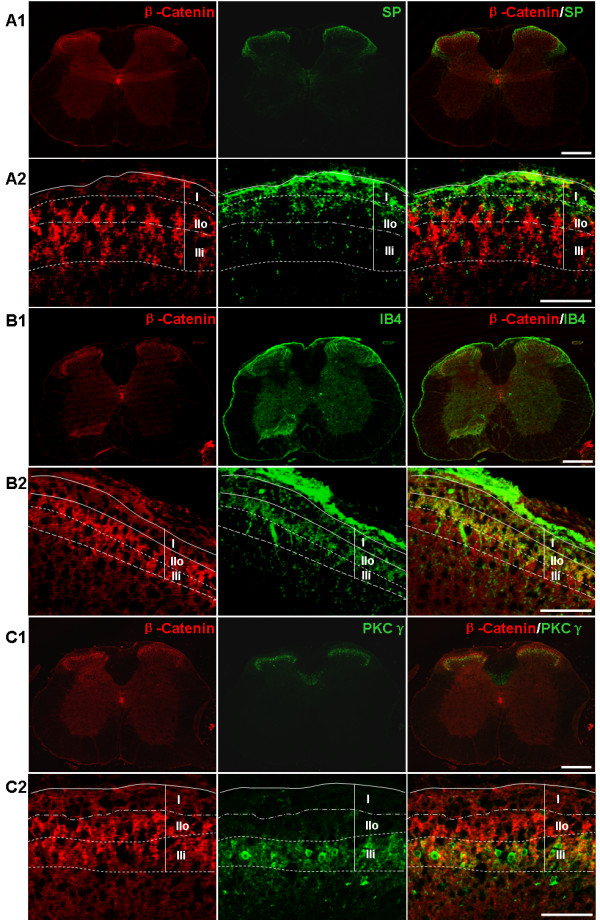
**Spatial distribution of β-catenin in normal mouse SCDH.A1**-**A2**: Double-staining of β-catenin and SP, a marker of lamina I and outer layer of lamina II. **A1**. Low power images. β-catenin is detected in the gray matter of the spinal cord. In the SCDH, β-catenin displays a predominant band. **A2**. Higher power images of the β-catenin and SP staining in the superficial layers of the SCDH. Relatively moderate levels of β-catenin staining are detected in SP-marked layer I (I). A band with intensive β-catenin staining is observed mainly in lamina II, which partially overlaps the SP-stained outer layer of lamina II (IIo). **B1**-**B2**. Double-staining of β-catenin and IB4, a marker of the outer layer of lamina II. **B1**. Low power images. **B2**. Higher power images of the β-catenin and IB4 staining in the superficial layers of the SCDH. The outer half of the β-catenin band overlaps with IB4 (IIo), while the inner half (IIi) does not. **C1**-**C2**: Double-staining of β-catenin and PKCγ, a marker of the inner layer of lamina II (IIi). **C1**. Low power images. **C2**. Higher power images of the β-catenin and PKCγ staining in the superficial layers of the SCDH. The inner half of the β-catenin band overlaps with PKCγ. Scale bar for A1, B1 and C1: 300 μm; Scale bar for A2, B2 and C2: 50 μm.

**Figure 2:**
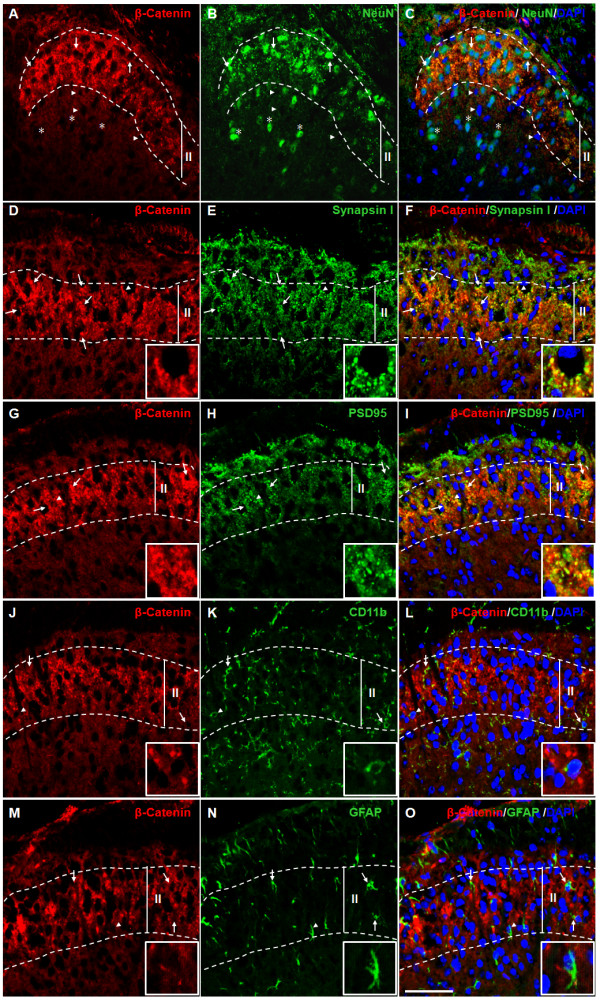
**Cellular localization of β-catenin in the SCDH.A**-**C**: Double-staining of β-catenin (**A**) and neuronal marker NeuN (**B**). Small clusters of β-catenin immunoreaction product (red) are in the cytoplasm around NeuN-labeled neuronal nuclei (arrows). Low levels of β-catenin staining are also observed around non-neuronal cell bodies (arrowheads) deep in the SCDH. Little staining is seen around NeuN in deep SCDH regions (asterisks). **D**-**F**: Double-staining of β-catenin (**D**) and synapsin I (**E**), a pre-synaptic marker. The β-catenin staining substantially overlaps with synapsin I detected in lamina II (**F**, arrows). **G**-**I**: Double-staining of β-catenin (**G**) and PSD95 (**H**). β-catenin staining overlapped with PSD95 staining (**I**, arrows). **J**-**L**: Double-staining of β-catenin (**J**) and CD11b (**K**). β-catenin was barely detectable in CD11b-postive microglial cells (**L**, arrows). **M**-**O**: Double-staining of β-catenin (**M**) and GFAP (**N**). β-catenin staining did not overlapped with GFAP staining (**O**, arrows). Insets are images at a higher magnification of the areas indicated by arrowheads, to show more clearly the spatial relation of β-catenin and various molecular markers. DAPI (blue) staining was performed to visualize all cells. Scale bar: 50 μm.

Because the Wnt/β-catenin pathway plays important roles in synaptic plasticity such as long-term potentiation (LTP) [22,33], we were interested in testing if this pathway is involved in the regulation of central sensitization. As an initial step toward this goal, we performed fluorescent immunostaining in naïve mice to determine the spinal distribution of β-catenin and Wnt3a, two signaling proteins in the canonical pathway. We observed that β-catenin immunostaining formed a predominant band in the dorsal horn, although a low level of signal was detected throughout the gray matter of the spinal cord (Figure 1). To define further the laminar distribution of the protein in the SCDH, we used molecular markers to label the specific layers of the dorsal horn. We found that β-catenin was enriched in lamina II, both the inner (IIi) and outer (IIo) segments (Figure 1), while its staining in lamina I was relatively low (Figure 1 A1-2). Staining for isolectin B4 (IB4) and PKCγ, considered as specific markers for the outer and inner segments of lamina II, respectively, confirmed the presence of β-catenin in both the lamina IIi and IIo (Figure 1 B1-2 and C1-2). These observations indicate that the β-catenin is enriched in lamina II of the SCDH. Previous studies revealed that β-catenin is expressed in hippocampal neurons [33]. We performed double-staining experiments, using NeuN to label neuronal cell bodies. As shown in Figure 2 A-C, label for β-catenin in lamina II was observed in regions surrounding neuronal nuclei labeled by NeuN, indicating that the majority of β-catenin was in neuronal cytoplasm. On the other hand, β-catenin staining in non-neuronal cell bodies (NeuN-negative; DAPI-labeled) was detectable but relatively low.

The β-catenin label was also clustered into small spots or dots (Figure 2). Because β-catenin is enriched in synapses [33], we next tested if the clustered β-catenin dots corresponded to synapses. To this end, we performed double-labeling experiments with β-catenin and synapsin I (pre-synaptic marker) or PSD95 (post-synaptic marker). We observed that β-catenin staining substantially overlapped with that of synapsin I or PSD95 (Figure 2 D-I). On the other hand, little β-catenin was observed in the CD11b-labeled microglia (Figure 2 J-L) or GFAP-labeled astrocytes (Figure 2 M-O). These results suggest an enrichment of β-catenin at synapses in the SCDH.

**Figure 3:**
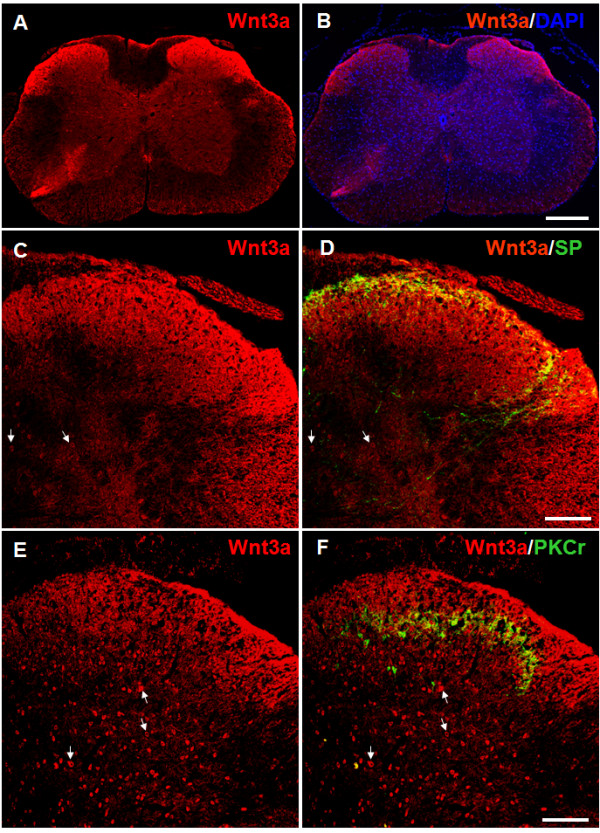
**Spatial distribution of Wnt3a in mouse SCDH.A**. Immunostaining of Wnt3a in mouse spinal cord. Wnt3a (red) is detected throughout the entire gray matter, with more intensive label in the superficial laminae of SCDH. **B**. A merged image of Wnt3a and DAPI signals to show the distribution of Wnt3a-positive cells. **C**-**D**. Wnt3a staining (**C**) and double-staining of Wnt3a and SP (**D**). Wnt3a staining is observed in the SP-labeled (green) lamina I layer and the deeper regions in the SCDH. **E**-**F**. Wnt3a staining (**E**) and double-staining of Wnt3a and PKCγ (**F**). Wnt3a staining is detected in PKCγ-labeled (green) lamina II and probably lamina III. Scattered cells with strong Wnt3a staining are also in the deep SCDH laminae (arrows, C-F). Scale bars for A and B: 300 μm; Scale bars for C-F: 100 μm.

**Figure 4:**
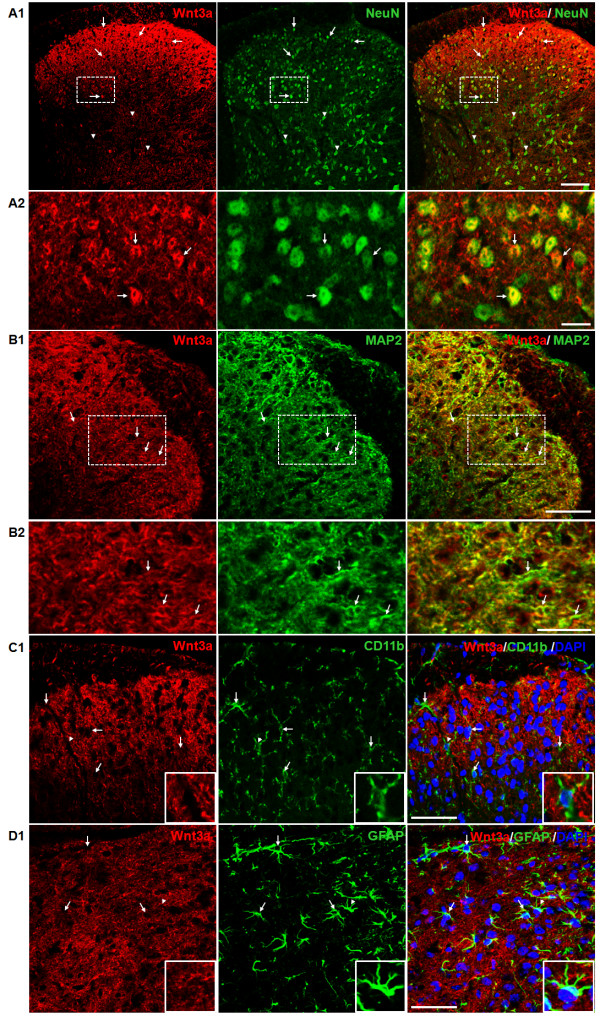
**Cellular localization of Wnt3a in the superficial laminae of the SCDH.A1**-**A2**. Double-staining of Wnt3a and NeuN. **A1**. Low power images. Wnt3a (red) is in NeuN-labeled neurons (green) (arrows). Some NeuN-positive cells have little Wnt3a signal (arrowheads). **A2**. Higher power images. **B1**-**B2**. Double-staining of Wnt3a and MAP2. **B1**. Low power images. Wnt3a staining (red) largely overlaps with MAP2 staining (green; arrows). **B2**. Higher power images. **C1**: Double-staining of Wnt3a and CD11b. Wnt3a (red) is not in CD11b (green)-labeled microglia (arrows). **D1**: Double-staining of Wnt3a and GFAP. Wnt3a (red) is not in GFAP (green)-labeled astrocytes (arrows). Insets in C1 and D1 are the higher-power images of the areas indicated by arrowheads. Scale bars for A1: 100 μm, A2: 20 μm, B1, C1 and D1: 50 μm, B2: 25 μm.

Next, we determined the spatial distribution of Wnt3a, a Wnt ligand that activates the canonical pathway. As shown in Figure 3 A-B, Wnt3a was detected throughout the dorsal horn, with the highest concentration in the superficial layers. In addition, some brightly stained profiles that are likely to be cell bodies in the gray matter were also detected. To determine the spatial distribution of Wnt3a, we double-labeled Wnt3a with SP or PKCγ. The results showed that Wnt3a signals were observed in regions labeled by both SP and PKCγ, indicating that Wnt3a is enriched in the laminae I and II (Figure 3 C-F). Relatively low levels of Wnt3a staining were also observed in deep SCDH layers (Figure 3 E-F). Previous studies demonstrated that Wnt3a was localized in the cell bodies and dendrites in hippocampal neurons [22,33,51]. Similarly, we found that double-staining experiments with NeuN showed that Wnt3a staining was found in NeuN-labeled cell bodies (Figure 4 A1-A2). In addition, the Wnt3a staining outside of cell bodies largely overlapped with that of MAP2, a dendritic marker (Figure 4 B1-B2). Wnt3a staining was not observed in the microglial cells (Figure 4 C1) or astrocytes (Figure 4 D1). The results suggest that Wnt3a protein is largely restricted to neurons, especially in their cell bodies and dendrites. Similarly, Wnt5a protein is also mainly expressed in neurons in the SCDH, while its co-receptor Ror2 in both neurons and astrocytes (submitted).

### Wnt3a and Wnt5a protein in mouse dorsal root ganglia (DRG)

**Figure 5:**
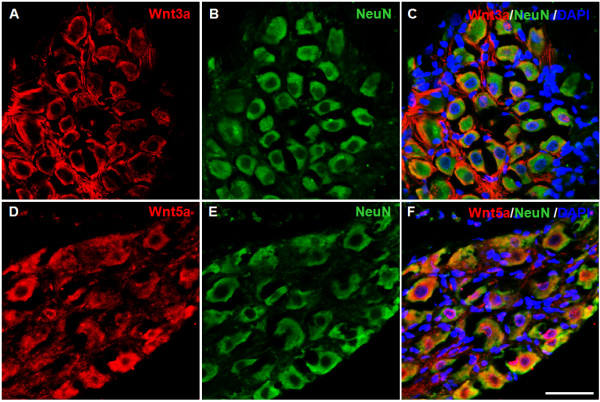
**Cellular localization of Wnt3a and Wnt5a in DRGs.A**-**C**: Double-staining of Wnt3a (**A**) and NeuN (**B**). Wnt3a is mainly detected in NeuN-labeled cells in DRGs (**C**). **D**-**F**: Double-staining of Wnt5a (**D**) and NeuN (**E**). Wnt5a is largely detected in NeuN-labeled DRG neurons (**F**). Scale bar: 50 μm.

We also determined the cellular localization of Wnt3a in DRGs. As shown in Figure 5 A-C, Wnt3a was expressed in NeuN-labeled neurons in DRGs (L4/L5 levels). Similarly, Wnt5a staining was also largely restricted to DRG neurons (Figure 5 D-F). Little Wnt3a or Wnt5a staining was detected in non-neuronal cells. Thus, Wnt3a and Wnt5a are expressed in neurons both in the spinal cord and the DRGs.

### Regulated expression of Wnt signaling proteins in the capsaicin pain model

**Figure 6:**
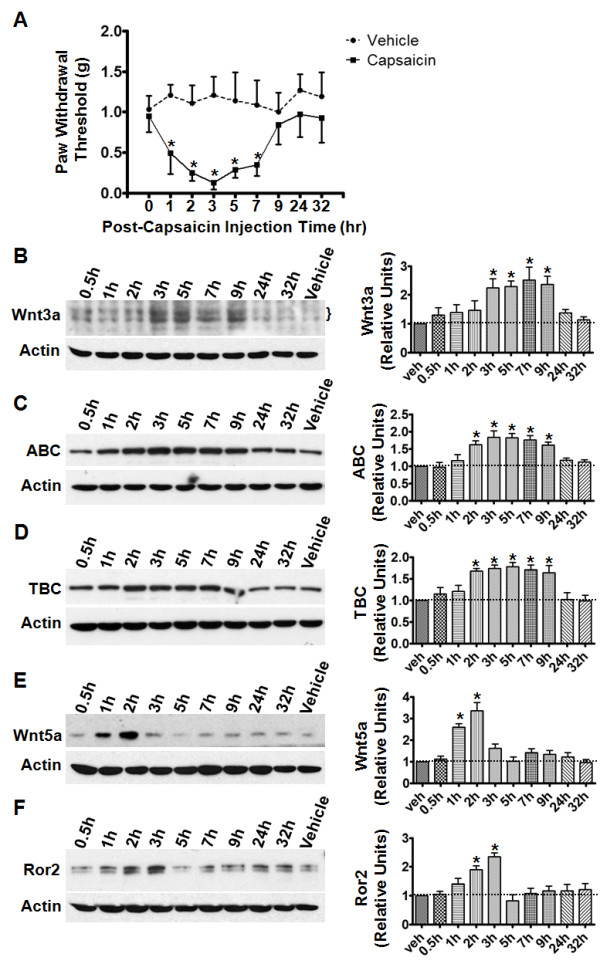
**Up-regulation of Wnt signaling proteins in the capsaicin pain model.A**. Time course of mechanical allodynia induced by intradermal (i.d.) capsaicin injection in mouse hind paws. Mice with saline injection were used as controls (vehicle). Following capsaicin administration, mechanical hypersensitivity peaked at 3 h post-injection and gradually returned to baseline (*, p < 0.05; n = 6). **B**-**D**. Protein levels of Wnt3a (**B**), active β-catenin (ABC, C), and total β-catenin (TBC, D) at different time points after capsaicin injection. Proteins levels gradually increased and peaked around 3 h after injection. **E**-**F**. Temporal expression profiles of Wnt5a (**E**) and Ror2 C (**F**) following capsaicin injection. The levels of both proteins transiently increased after the injection. β-actin was included as a loading control. In the summary graphs (right panels), protein levels from at least three independent experiments are presented as relative units to the vehicle controls (mean ± SEM; *, p < 0.05; one-way ANOVA).

The expression of Wnt3a and β-catenin in SCDH superficial layers suggests a potential role of Wnt signaling in nociceptive processing. Thus, we sought to determine whether peripheral painful stimulation affected the expression of the Wnt signaling proteins in SCDH. We first employed the capsaicin pain model, created by intradermal (i.d.) injection of capsaicin in hind paw [52]. It is well established that this pain model develops central sensitization [50,53,54]. Following capsaicin administration, mice developed mechanical hypersensitivity demonstrated by a decrease in paw withdrawal threshold (PWT) (Figure 6 A). The mechanical allodynia was observed at 1 h after capsaicin injection, and it peaked at 3 h. After 7 h post-injection, the mechanical sensitivity gradually decreased and PWT returned to normal. Results of immunoblotting showed that Wnt3a, active β-catenin (ABC) and total β-catenin (TBC) increased in the SCDH during the period of increased mechanical sensitivity (Figure 6 B-D). Consistent with the assumption that Wnt3a and β-catenin are in the same (canonical) pathway, Wnt3a, ABC and TBC proteins followed similar temporal profiles of up-regulation. The protein levels started to increase at 1 h after capsaicin injection and peaked at 3–5 h. Furthermore, significantly higher levels of these proteins were still observed at 9 h (Figure 6 B-D). The magnitude of increase differed for each protein: Wnt3a peaked at ~2.5 fold increase whereas ABC or TBC peaked at ~1.8 fold increase. Although cautions were taken to avoid potential contamination of the dorsal horn tissues from DRGs and dorsal root fibers, we anticipate that there were still peripheral fibers intermingling in the dissected dorsal horn. Thus, although it is likely that the observed up-regulation of Wnt signaling proteins was mainly contributed by the dorsal horn cells, we cannot exclude the possibility that the up-regulation also occurred in peripheral sensory neurons.

In addition, we also examined the effect of capsaicin-induced pain on proteins in the non-canonical pathways. We focused here on Wnt5a, a prototypic Wnt ligand that activates the non-canonical pathways. As shown in Figure 6 E, Wnt5a was also induced in the SCDH following i.d. injection of capsaicin. The temporal profile of capsaicin-induced Wnt5a alteration differed from that of Wnt3a and β-catenin. The Wnt5a up-regulation peaked at 2 h after capsaicin injection, but returned to baseline by 3 h (Figure 6 E). These data indicate that capsaicin up-regulates Wnt5a in a more rapid and transient manner. Furthermore, we also examined the temporal profile of Ror2, a Wnt5a receptor tyrosine kinase that activates JNK signaling [55]. Similar to Wnt5a, Ror2 was also transiently up-regulated (Figure 6 F). Compared with Wnt5a, the Ror2 up-regulation was delayed by 1 h (Figure 6 F). The overlapping but distinct temporal profiles of Wnt5a and Ror2 indicate that Wnt5a does not solely depend on Ror2 to transmit signals.

### Regulated expression of Wnt signaling proteins in the HIV-gp120 pain model

**Figure 7:**
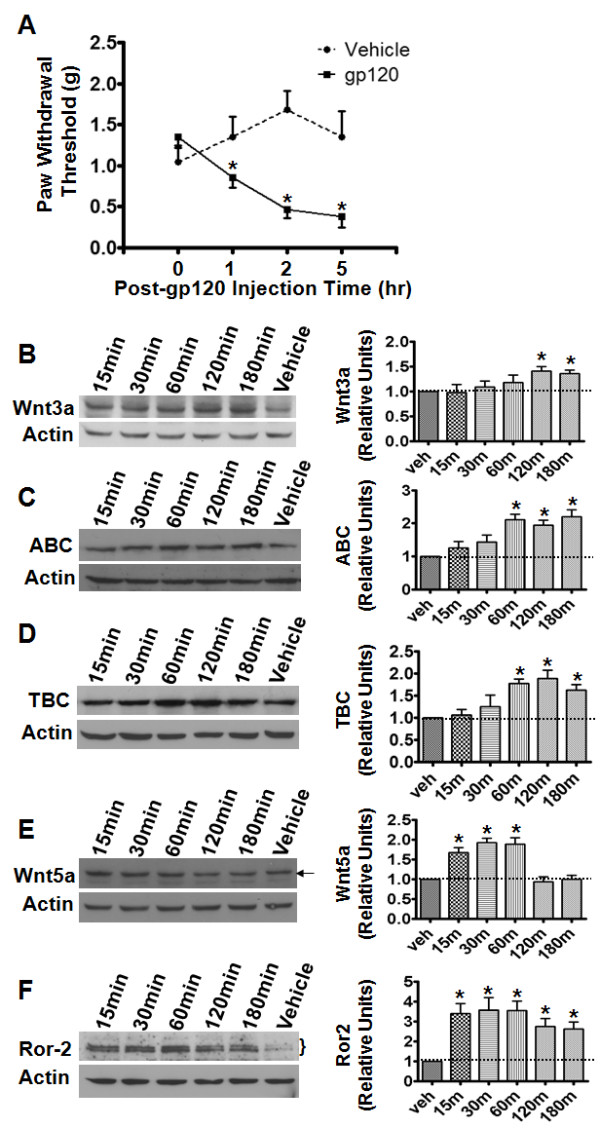
**Expression profiles of Wnt signaling proteins in the HIV-gp120 pain model.A**. Time course of mechanical allodynia induced by intrathecal (i.t.) HIV-gp120 injection (vehicle controls: saline injection). Following gp120 administration, mice developed mechanical hypersensitivity. **B**-**D**. Protein levels of Wnt3a (**B**), ABC (**C**), and TBC (**D**) at different time points after HIV-gp120 injection. The proteins were gradually up-regulated after the injection. **E**-**F**. Expression of Wnt5a (**E**) and Ror2 (**F**) proteins at different time points after gp120 injection. Wnt5a rapidly increased, peaked at 15–30 min after injection, and returned to baseline by 2 h. Ror2 was also up-regulated and persisted at a high level at 3 h after injection. Data from at least three independent experiments are summarized in graphs at right (*, p < 0.05; one-way ANOVA).

We next determined the regulated expression of Wnt proteins in the HIV gp120 pain model. Previous work established that intrathecal injection (i.t.) of HIV-gp120 protein induces hyperalgesia and mechanical allodynia in animals [56-59]. Indeed, following gp120 administration, mice showed a progressive decrease in PWT evoked by von Frey filaments (Figure 7 A). The mechanical allodynia was observed at 1 h after gp120 injection, and fully developed by 2–5 h. Immunoblotting results showed that Wnt3a, ABC and TBC progressively increased in the SCDH during the development of allodynia (Figure 7 B-D). The protein levels started increasing within 1 h after gp120 injection and peaked at 2–3 h (Figure 7 B-D). Although the magnitudes of the peak increases differed among Wnt3a (1.4 fold, *p* < 0.05), ABC (2.2 fold, *p* < 0.05) and TBC (1.9 fold, *p* < 0.05), the proteins displayed similar temporal profiles of up-regulation. The progressive up-regulation of the proteins seemed to be parallel to the temporal profile of allodynic expression (Figure 7 A).

We also examined the expression profiles of Wnt5a and Ror2 in the gp120 pain model. The results showed that Wnt5a rapidly increased and peaked at 15–30 min after gp120 injection (Figure 7 E). Similar to the Wnt5a expression in the capsaicin pain model (Figure 6 E), the up-regulation of Wnt5a was relatively transient and returned to baseline by 2 h (Figure 7 E). Like Wnt5a, Ror2 also rapidly increased and peaked at 15–30 min after gp120 injection (Figure 7 F). Unlike Wnt5a, expression levels of Ror2 were maintained at significantly higher levels over baseline for 3 h (Figure 7 F). In addition, the magnitude of the Ror2 increase (3.6 fold) was higher than that of Wnt5a (1.9 fold).

### Regulated expression of Wnt signaling proteins in the neuropathic pain model

**Figure 8:**
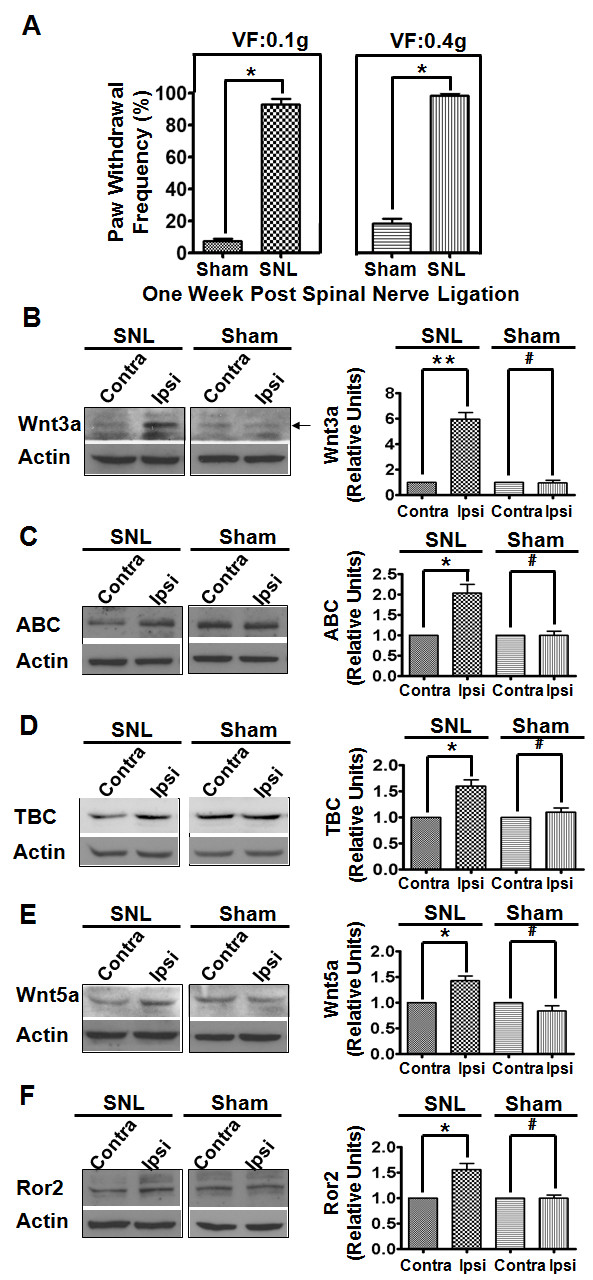
**Up-regulation of Wnt signaling proteins in the neuropathic pain model.A**. Neuropathic pain was induced 1 week post L5 spinal nerve ligation (SNL, n = 6). Mice with sham operation (without SNL, n = 6) were used as controls. **B**-**F**. Wnt3a (**A**), ABC (**B**), TBC (**C**), Wnt5a (**D**), and Ror2 C (**E**) proteins in the ipsilateral (ipsi) and contralateral (contra) sides of the SCDH at 7 days after unilateral L5 spinal nerve ligation (SNL). Compared with the contra side, significant increases in the levels of Wnt signaling proteins were detected in the ipsi side of SNL but not control mice (n = 3). Data are summarized in graphs at right (**, p < 0.01; *, p < 0.05; #, p > 0.05; student’s t-test).

Next, we were interested in examining the regulatory effect induced by peripheral nerve injury on Wnt signaling proteins. In this experiment, we used the neuropathic pain model produced by unilateral L5 spinal nerve ligation (SNL) [60], which is a well-established model that develops various hallmarks of chronic pain and central sensitization including neuroinflammation, hyperexcitation of spinal dorsal neurons and disinhibition of inhibitory interneurons [61-63]. As shown in Figure 8A, one week after SNL, the mice demonstrated increased paw withdrawal frequencies in response to mechanical stimulation with von Frey filaments: 0.10 g force, 92.86 ± 3.59% compared to 7.15 ± 1.84% (*p* < 0.05, n = 6) and 0.40 g force, 98.57 ± 1.43% compared to 18.57 ± 2.6% (*p* < 0.05, n = 6), for the sham-operated mice. Immunoblotting analysis of the SCDH from SNL mice at one week post-ligation showed that Wnt3a was significantly up-regulated in the ipsilateral (ipsi) compared to the contralateral (contra) side (5.9 fold, *p* < 0.01) (Figure 8 B). Similarly, both ABC (Figure 8 C) and TBC (Figure 8 D) were increased in the ipsi side of the SCDH with similar magnitudes of increase (2.0 fold, *p* < 0.05 for ABC and 1.6 fold, *p* < 0.05 for TBC). In addition, we also observed that non-canonical pathway signaling proteins, Wnt5a (Figure 8 E) and its co-receptor Ror2 (Figure 8 F), increased in the SNL model. Thus, Wnt signaling proteins are up-regulated following peripheral nerve injury.

## Discussion

We describe here the expression of Wnt signaling proteins in the SCDH and their change in expression in three pain models. We show that β-catenin is enriched in neurons in lamina II, and that Wnt3a is abundant in neurons in the superficial layers (laminae I-III). We also show that these and other Wnt signaling proteins (Wnt5a and Ror2) are up-regulated in the SCDH in these pain models. Our data suggest potential involvement of Wnt signaling pathways in the regulation of central sensitization in acute and chronic pain. Future studies are warranted to directly test this hypothesis.

Wnt signaling may contribute to chronic pain via multiple routes. We found that β-catenin is enriched in SCDH lamina II, especially at synaptic regions. Lamina II neurons, which include both excitatory and inhibitory interneurons, play crucial roles in central sensitization [48,64,65]. Because β-catenin is known to regulate synaptic transmission, synapse/spine assembling and remodeling [38,39], the observation of enriched β-catenin protein at the synapses in lamina II suggests that canonical Wnt/β-catenin signaling may regulate synaptic plasticity in the neurocircuitry processing nociceptive input in the SCDH. Consistent with its role in central sensitization, β-catenin is up-regulated in capsaicin, HIV gp120, and SNL pain models. While we found β-catenin is up-regulated at 7 days after SNL, a recent study showed that this protein significantly increases at 1 and 3 days and returns to baseline at 7 days in the rat SCDH after unilateral spared nerve injury (SNI) [66]. These findings indicate that the regulated expression of β-catenin in the SCDH of different neuropathic pain models follows different temporal patterns. In further support of a role of β-catenin signaling, Wnt3a, a prototypic Wnt ligand for the canonical Wnt/β-catenin pathway, is also expressed in the superficial laminae (including lamina I) and is up-regulated in these pain models. Previous studies show that activation of NMDA receptors by synaptic stimulation elicits Wnt3a secretion from hippocampal synapses to activate β-catenin signaling and facilitate long-term potentiation [33]. One may conceive that activation of NMDA receptors in the SCDH by nociceptive stimuli could also cause Wnt3a secretion to facilitate central sensitization via β-catenin. Signaling proteins in the non-canonical pathway, including Wnt5a and Ror2, are also up-regulated in the pain models. Recent studies have shown that Wnt5a is an NMDAR-regulated protein [34] and critical for the differentiation and plasticity of excitatory synapses [21,23]. Ror2 may mediate the activity of Wnt5a in the regulation of synapse differentiation [67]. In addition, Wnt5a also regulates GABA receptor recycling at inhibitory synapses [68]. These previous findings suggest that the observed up-regulation of Wnt5a and Ror2 in these pain models may also contribute to synaptic remodeling during the development of chronic pain.

Neuroinflammation in the SCDH is a constant manifestation of chronic pain in animal models. Pro-inflammatory factors such as IL-6, IL-1β, TNF-α and MCP-1 play important roles in the initiation and maintenance of chronic pain [11,15,69]. Recent studies have suggested that Wnt5a signaling may regulate the peripheral inflammatory response in chronic disorders, including sepsis [70], rheumatoid arthritis [71], atherosclerosis [72], melanoma [73], and psoriasis [74]. Wnt5a is known to activate CaMKII signaling to modulate the macrophage-mediated inflammatory response [70]. Our previous studies revealed that Wnt5a evokes the expression of proinflammatory cytokines (IL-1β and TNF-α) in primary cortical cultures, indicating a role of Wnt5a in the regulation of neuroinflammation in CNS [75]. Wnt5a is up-regulated by i.t. gp120 and peripheral nerve injury, and each of these is known to induce persistent neuroinflammation in SCDH [76-78]. We propose that one potential mechanism by which up-regulated Wnt5a may facilitate chronic pain development is by promoting neuroinflammation.

The temporal expression of proteins in canonical and non-canonical pathways appears to follow differential profiles after pain induction. β-catenin and Wnt3a, which are in the canonical pathway, displayed a gradual increase following capsaicin or HIV-gp120 administration. Their gradual up-regulation is correlated with the progressive development of capsaicin-induced and gp120-induced mechanical allodynia and stays at a peak level when mechanical sensitivity starts decreasing. On the other hand, Wnt5a and Ror2 in the non-canonical pathway showed a more rapid but transient increase; their up-regulated expression came back to baseline when capsaicin-induced allodynia was still at a maximal level. These observations suggest that the canonical and non-canonical Wnt signaling pathways may have distinct biological functions in different phases of chronic pain development.

## Materials and methods

### Experimental animals

Young adult male C57 BL/6 J mice (8–10 weeks), purchased from Jackson Laboratory (Bar Harbor, Maine, USA) were used for all studies. Animals were housed in a constant-temperature environment with soft bedding and free access to food and water under a 12/12-h light–dark cycle. All animal procedures were performed in accordance with an animal protocol approved by the Institutional Animal Care and Use Committee at the University of Texas Medical Branch (protocol #: 0904031) and adhered to the guidelines of the International Association of the Study of Pain for the ethical care and use of laboratory animals [79].

### Capsaicin pain model

The mouse capsaicin pain model was generated as described [52]. Briefly, mice were anesthetized with isoflurane (2% for induction and 1.5% for maintenance) in a flow of O_2_ and placed in a prone position. For each mouse, 5 μl of capsaicin (0.5% in saline containing 20% alcohol and 7% Tween 80; purchased from Sigma) was injected intradermally (i.d) into the plantar region of hind paw using a 30 gauge needle attached to a Hamilton Syringe. Mice injected with vehicle were used as controls. Five minutes later, injected mice were returned to their home cages.

### HIV-gp120 pain model

The recombinant HIV-gp120 protein (HIV Bal gp120; NIH AIDS Research and Reference Reagent Program) in PBS was stored in a −80°C freezer. At the time of injection, gp120 was slowly thawed, diluted to a concentration of 20 ng/μl in ice-cold PBS and maintained on ice. For gp120 administration, mice were anesthetized under 2% isoflurane, and 5 μl gp120 (100 ng) was intrathecally (i.t) injected into the subarachnoid space between the L5 and L6 vertebrae using a 30 gauge needle attached to a Hamilton Syringe [57,80]. Mice injected with vehicle were used as controls.

### Neuropathic pain model

Peripheral neuropathy in mice was produced by a unilateral L5 spinal nerve ligation as previously reported [60,81]. Briefly, mice were anesthetized with 2% isoflurane, and the left L5 spinal nerve was isolated and tightly ligated with 7-0 silk thread. Mechanical sensitivity was assessed 7 days after ligation.

### Immunohistochemistry

Adult mice were deeply anesthetized with 4% isoflurane and perfused transcardially with 50 ml of D-PBS, followed by 50 ml of paraformaldehyde (PFA; 4% in 0.1 M phosphate buffer). The L4 and L5 DRG, and lumbar spinal cord tissues were dissected out, post-fixed in the same PFA solution for 3 hr at 4°C, and then cryoprotected in sucrose solution (30% in 0.1 M phosphate buffer) overnight at 4°C. Transverse sections (15 μm) were prepared on a cryostat (Leica CM 1900) and thaw-mounted onto Superfrost Plus microscope slides. For immunostaining, sections were incubated in blocking buffer (5% BSA and 0.3% Triton X-100 in 0.1 M phosphate buffer) for 1 h at room temperature, followed by overnight incubation with anti-β-catenin (1:500, BD: 610153), anti-substance P (SP, 1:1000, Abcam: ab10353), anti-IB4 (1:400, Sigma: L2895), anti-PKCγ (1:1000, Santa Cruz: SC211), anti-NeuN (1:200, Millipore: MAB377), anti-MAP2 (1:400, Millipore: MAB378), anti-Wnt5a (1:200, Abcam: ab72583), anti-Synapsin I (1:400, Millipore: AB1543), anti-PSD95 (1:400, Cell Signaling: 2507), anti-GFAP (1:500, Millipore: 04-1062 and MAB360), anti-CD11b (1:100, AbD: MCA74GA) or Wnt3a (1:200, Millipore: 09162) antibody. After five washes with PBS (0.1 M phosphate buffer), the sections were incubated with FITC or Cy3-conjugated secondary antibody (1:200, Jackson ImmunoResearch Laboratories), followed by incubation with DAPI (Sigma). IgG from the same animal sources was used as negative controls for immunostaining. Images were captured using a laser confocal microscope (Zeiss).

### Western blotting analysis

Mice were anesthetized and sacrificed and the L4-L6 lumbar spinal cord segments were collected. The dorsal halves were dissected on an ice-chilled plate, and the dorsal roots were cut off under dissecting microscopes. The collected dorsal spinal tissues were homogenized in RIPA lysis buffer (1% Nonidet P-40, 50 mM Tris–HCl, pH 7.4, 1% sodium deoxycholate, 150 mM NaCl, 1 mM EDTA, pH 8.0) with a protease inhibitor cocktail (Sigma). Equal amounts of protein were loaded and separated by SDS-PAGE. Protein was transferred to pure nitrocellulose membranes, which were then blocked and incubated with anti-total-β-catenin (TBC, 1:5000, BD: 610153), anti-Active-β-catenin (ABC, 1:1000, Millipore: 05665), anti-Wnt3a (1:1000), anti-Wnt5a (1:1000), or anti-Ror2 (1:1000, a gift form Dr. Roel Nusse, Stanford University School of Medicine [55] ) primary antibodies. Protein bands were visualized by an Enhanced Chemiluminescence kit (Pierce).

### Mechanical allodynia

For the capsaicin and gp120 pain models, a series of calibrated von Frey filaments (0.1 to 2.0 g) were applied to the plantar surface of the mouse hind paw using the “up and down paradigm” described previously [82,83]. Mechanical allodynia was assessed by changes in paw withdrawal threshold in response to von Frey stimuli. For the SNL neuropathic pain model, mechanical sensitivity was assessed before and seven days after ligation by paw withdrawal frequencies in response to von Frey stimuli as previous reported [60,81].

### Data analysis and statistics

Densitometry of Western blotting was conducted and quantified using the ImageJ software (NIH) with β-actin as the loading control. Values were represented as mean ± SEM of 3 separate experiments. Statistical analysis was performed using Prism 5 (GraphPad) software. One-way ANOVA or student’s t-test was used to analyze data from different groups. Two-way repeated measures ANOVA with one repeated factor (time) was used for mechanical threshold data analysis (p < 0.05 was considered significant).

## Competing interests

The authors declare that they have no competing interests.

## Authors’ contributions

YS, SY, BL, and JW carried out experiments and data analyses. ST, YS, SC, KC, and JC designed the study and prepared the manuscript. All authors read and approved the final manuscript.
